# Supplementary material for: Causal Relationship between Aging and Anorexia Nervosa: A White-Matter-Microstructure-Mediated Mendelian Randomization Analysis
Source: Biomedicines. 2024 Aug 16;12(8):1874. doi: 10.3390/biomedicines12081874 (PMC11351342; doi:10.3390/biomedicines12081874)
Supplement: Supplementary file 1 [file biomedicines-12-01874-s001.zip › Supplementary Figure/Figure S4/Singe SNP plot of MR analysis for TL to ubm-b-1964.pdf]

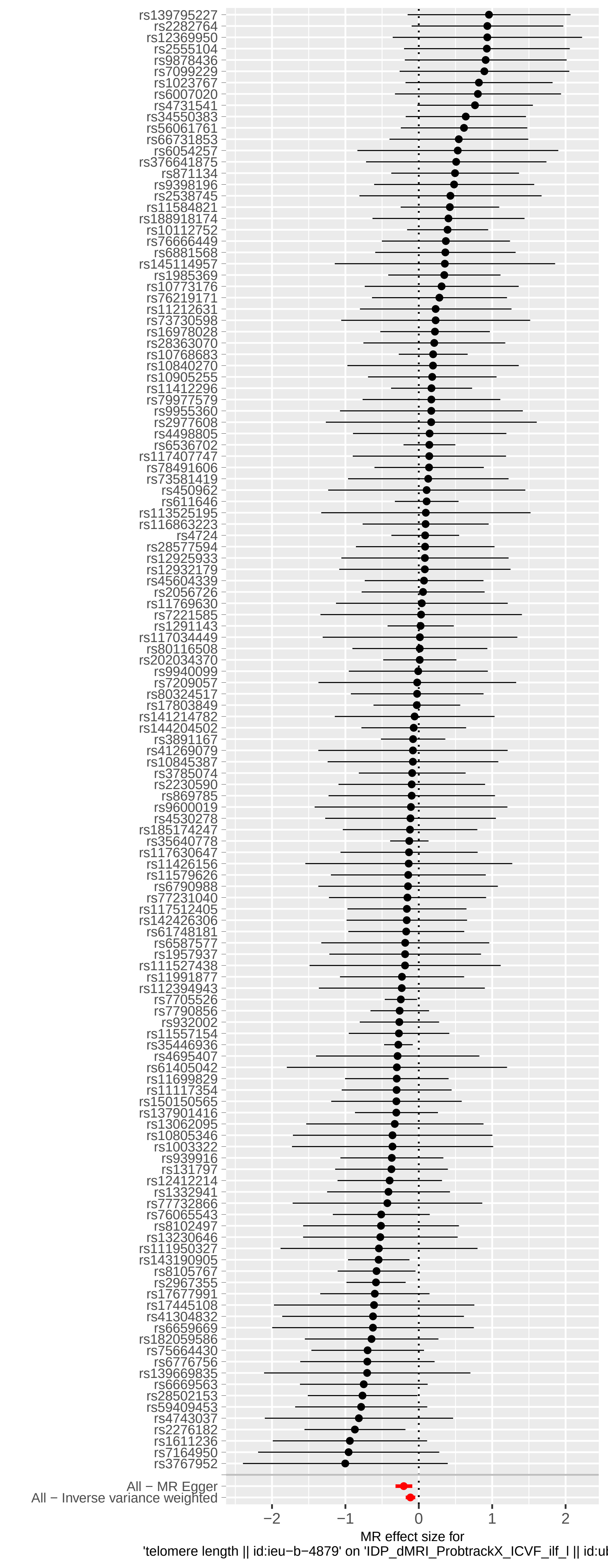
rs139795227
rs2282764
rs12369950
rs2555104
rs9878436
rs7099229
rs1023767
rs6007020
rs4731541
rs34550383
rs56061761
rs66731853
rs6054257
rs376641875
rs871134
rs9398196
rs2538745
rs11584821
rs188918174
rs10112752
rs76666449
rs6881568
rs145114957
rs1985369
rs10773176
rs76219171
rs11212631
rs73730598
rs16978028
rs28363070
rs10768683
rs10840270
rs10905255
rs11412296
rs79977579
rs9955360
rs2977608
rs4498805
rs6536702
rs117407747
rs78491606
rs73581419
rs450962
rs611646
rs113525195
rs116863223
rs4724
rs28577594
rs12925933
rs12932179
rs45604339
rs2056726
rs11769630
rs7221585
rs1291143
rs117034449
rs80116508
rs202034370
rs9940099
rs7209057
rs80324517
rs17803849
rs141214782
rs144204502
rs3891167
rs41269079
rs10845387
rs3785074
rs2230590
rs869785
rs9600019
rs4530278
rs185174247
rs35640778
rs117630647
rs11426156
rs11579626
rs6790988
rs77231040
rs117512405
rs142426306
rs61748181
rs6587577
rs1957937
rs111527438
rs11991877
rs112394943
rs7705526
rs7790856
rs932002
rs11557154
rs35446936
rs4695407
rs61405042
rs11699829
rs11117354
rs150150565
rs137901416
rs13062095
rs10805346
rs1003322
rs939916
rs131797
rs12412214
rs1332941
rs77732866
rs76065543
rs8102497
rs13230646
rs111950327
rs143190905
rs8105767
rs2967355
rs17677991
rs17445108
rs41304832
rs6659669
rs182059586
rs75664430
rs6776756
rs139669835
rs6669563
rs28502153
rs59409453
rs4743037
rs2276182
rs1611236
rs7164950
rs3767952
All − MR Egger
All − Inverse variance weighted
−2
−1
0
1
2
MR effect size for
'telomere length || id:ieu−b−4879' on 'IDP_dMRI_ProbtrackX_ICVF_ilf_l || id:ul
